# Slovenian Nursing Students’ Perspectives and Experiences with Nursing Care of People with Intellectual and Developmental Disabilities: A Qualitative Descriptive Study

**DOI:** 10.3390/healthcare13010061

**Published:** 2025-01-01

**Authors:** Ljubiša Pađen, Renata Vettorazzi, Mirjam Ravljen, Manca Pajnič

**Affiliations:** Division of Nursing, Faculty of Health Sciences, University of Ljubljana, 1000 Ljubljana, Slovenia; ljubisa.paden@zf.uni-lj.si (L.P.); renata.vettorazzi@zf.uni-lj.si (R.V.); mirjam.ravljen@zf.uni-lj.si (M.R.)

**Keywords:** student, experience, adjustment, learning disabilities, content analysis

## Abstract

**Background/Objectives:** Providing nursing care for individuals with intellectual and developmental disabilities can be challenging for nursing students, often perceived as stressful, demanding, and, at times, unpleasant. This study aimed to describe the experiences of students in their interactions and provision of nursing care for people with intellectual and developmental disabilities. **Methods**: A qualitative descriptive study was conducted. Fourteen self-reflections from nursing students were analysed by using qualitative content analysis. **Results**: The findings indicate that students experience contact and nursing care for individuals with intellectual and developmental disabilities as dynamic and varied. This process is characterised by transitions in communication and interaction, emotional transitions, and transitions in knowledge and understanding of caring and nursing care. **Conclusions**: Students encountered emotional, cognitive, and behavioural challenges when interacting with and providing care for individuals with intellectual and developmental disabilities. Their experience is described as multidimensional and demanding. Gaining insight into these experiences and understanding the students’ perspectives are essential for planning future theoretical and practical nursing education.

## 1. Introduction

Intellectual and developmental disabilities (IDDs) are defined as disorders that arise during the developmental period (before the age of 18) and are characterised by deficits in intellectual abilities and impaired adaptive functioning [1]. Research shows that people with IDDs are more frequently hospitalised and have higher rates of complications from chronic illnesses compared with individuals without IDDs [2,3], which often leads to more frequent contact with the healthcare system. They are frequently cared for by healthcare professionals who are not specifically trained in working with individuals with special needs, and the complexity of care often makes working with individuals with IDDs a source of stress for these professionals [4,5].

Nurses and nursing students often hold negative attitudes and uncomfortable feelings towards people with IDDs. The reasons for such attitudes and feelings are associated with cognitive, behavioural, and affective factors, such as prejudice, stereotypes, fears, limited interaction, and others [6,7,8,9,10]. Research findings also indicate that healthcare professionals form positive attitudes if they have prior experience with individuals with IDDs [11,12]. Additionally, a study conducted by Karl et al. [13] showed that an appropriate educational programme preparing students for work with individuals with IDDs increases the development of positive attitudes towards people with IDDs.

A smaller number of studies examined the experiences and perceptions of nurses in caring for individuals with IDDs [4]. The same authors found that nurses do not feel sufficiently prepared to provide care for people with IDDs. It is also notable that nursing students report they would not choose to work in the area of caring for individuals with IDDs [14], which may have significant implications for the care of people with IDDs and for the future planning of healthcare services.

In Slovenia, historically, people with IDDs have been placed in specialised institutions established in the 1950s [15]. Initial attempts at deinstitutionalisation began in the late 1960s; however, the process remains incomplete to this day, and people with IDDs still lack opportunities for individualised and personalised care. The process of deinstitutionalisation (which is not limited to providing alternative living arrangements, such as independent and autonomous living, but also includes the deinstitutionalisation of education, the establishment of relevant legislation, and more) has primarily involved professionals such as social workers, psychologists, and special and social educators [16,17] with less involvement from nursing professionals, who have throughout the past practised within the biomedical model rather than the biopsychosocial model of health [18].

In Slovenia, nursing education is provided at two levels: secondary level education (nursing assistants/associates and pre-university education, lasting 4 years) and higher education (1st cycle degrees and professional studies, lasting 3 years) [19]. The latter is regulated by the European Directives [20,21]. Higher education programmes include topics related to nursing care for individuals with IDDs as part of mandatory courses but not as a dedicated subject. Additionally, there are no specialisations or postgraduate programmes focusing on people with IDDs. In contrast, education on IDD nursing is already available at the undergraduate level in the UK [22] and postgraduate level in Ireland [23].

There are also differences between Slovenia and north-western EU countries in healthcare practices. Specifically, in Slovenia, the majority of care is still delivered in institutions rather than communities. Additionally, there are no specialised community nursing services for people with IDDs. Furthermore, there are generally long waiting times and a lack of access to services, such as therapies and psychological care [24]. Another aspect is also general social structures affecting people with IDDs, such as social attitudes towards people with IDDs, which remain a barrier to their inclusion. There is still a stigma associated with people with IDDs, and societal support for their rights and inclusion is limited [17,25].

Findings from foreign studies are only partially transferable, as educational systems, healthcare practices, and social structures differ from those in Slovenia. Therefore, the aim of this study was to describe the experiences and perceptions of Slovenian students in providing nursing care to individuals with IDDs. The objective of this research was to answer the questions of how students perceived their interactions with individuals with IDDs and what their experiences were in delivering nursing care to individuals with IDDs.

## 2. Materials and Methods

### 2.1. Study Design

A qualitative descriptive study was carried out, as it allowed for a detailed, in-depth understanding of phenomena or experiences [26]. Due to the exploratory and descriptive nature of the research question, this study was based on a descriptive approach [27]. This design is particularly suitable when the aim is to provide a straightforward and comprehensive summary of participants’ experiences and perceptions [27,28] while staying close to the data and maintaining a low level of interpretation [29]. Unlike other qualitative approaches, such as phenomenology or grounded theory, qualitative descriptive studies focus on describing the “what” and “how” of a particular experience rather than generating theories or uncovering underlying meanings [26,27,28]. Qualitative research is valuable in healthcare research, where understanding participants’ experiences can inform practice, education, and policy development. This approach prioritises the participants’ voices and ensures that findings remain grounded in their perspectives, offering practical and meaningful insights into real-world issues [26,27,28,29].

### 2.2. Participants

A purposive sample was used in this study to explore students’ perceptions and experiences. Participants were included in this study if they were enrolled as second-year students in an undergraduate nursing programme during the 2019/2020 or 2020/2021 academic years, had completed the obligatory 15 h of theoretical training in nursing care for people with IDDs during their first year of study, and were undertaking clinical placements in nursing care for people with IDDs in their second year of study. A total of 35 potential participants were invited to take part. All others were excluded.

In Slovenia, the general nursing programme (a first-cycle degree; professional studies) is regulated by EU directives and spans three academic years. During their studies, nursing students are required to complete theoretical and at least 2300 h of clinical instruction in nursing related to general and specialist surgery, general and specialist medicine, community (home) care, geriatric care, paediatric care, obstetrics and gynaecology, and mental health and psychiatry [20,21].

### 2.3. Data Collection

Data were collected between October 2019 and May 2021 through unstructured written self-reflections from nursing students following the completion of a forty-hour clinical placement. During this placement, the students participated in assessing, planning, delivering, and evaluating nursing care for individuals with mild to moderate or moderate to severe IDDs, under the supervision of a registered nurse. Self-reflection enables one to examine their professional practice—events, actions, experiences, and perceptions—and to identify limitations in their approach, as well as aspects that may otherwise go unnoticed, with the aim of improving future practice [30,31]. Self-reflection is also frequently used as a data source in qualitative research, often in the form of essays [32] or diary entries [33,34,35].

In this study, participants were asked to reflect on two questions during their clinical placement related to nursing care for people with IDDs, namely, how they perceived their interactions with individuals with IDDs, and what their experience of delivering nursing care to these individuals was like.

### 2.4. Data Analysis and Rigour

A total of 14 students participated in this study and submitted self-reflections. The reflections were collated into a single document and analysed using content analysis [36]. All reflections were initially read to gain an overall impression of the quality of the collected data, followed by the decontextualisation of individual self-reflections into meaning units. This was then followed by recontextualisation, with coding and the development of categories supported by quotations. The initial analysis was carried out by the first author (L.P., male, a nurse by background and with experience in qualitative research methods). This was subsequently followed by a review, during which the analysis was discussed, and interpretation was agreed upon by all authors (R.V., M.R., and M.P., all females, and also nurses by background). An example of the analytical process is provided in Table 1.

To ensure the credibility of this study and its findings, the authors followed the principles described by Graneheim and Lundman [36]. Credibility was achieved through precise and transparent reporting, including a description of the research process and findings, along with participant quotes that reflected the subcategories and categories formed. Consistency was ensured through the data collection technique (all participants responded to the same prompts) and stable analysis (consistently assigning the same codes to the same meaning units).

The authors acknowledged the existence of multiple interpretations of reality but adhered to the most probable one, with the analysis focusing on the explicit content of the students’ reflections, without delving into hidden or latent content, thereby strengthening the credibility of the findings.

The Standards for Reporting Qualitative Research [37] guided the reporting in this study.

### 2.5. Ethics

Basic principles of ethics guided this study, namely, autonomy, beneficence, non-maleficence, and justice [38]. Participants were informed about the purpose of this study and their role if they chose to participate. They were informed that participation was voluntary, that they would receive no incentives, and that they could withdraw at any point before submitting their reflection. By submitting their self-reflections, participants agreed that the data could be used for research purposes.

Demographic data were not collected, thus ensuring complete anonymity. It was believed that anonymity would encourage participants to reflect on any potential negative feelings and attitudes towards individuals with IDDs, rather than reporting socially desirable or expected attitudes.

## 3. Results

The description of students’ perspectives and experiences with interacting and providing nursing care to individuals with IDDs was summarised through the following categories and subcategories: transitions in communication and relationships with individuals with IDDs (establishing contact and communication), transitions in emotions (feelings, empathy, and a need for relief in the relationship and experience), and transitions in knowledge and understanding of care and nursing care (knowledge transitions, nursing content, and conceptions of care and nursing care).

Quotes from multiple participants are included throughout the results to illustrate the richness and variety of perspectives. Due to the nature of the data collection method and the sensitivity of the topic, individual demographic information was not collected. This approach aimed to avoid socially desirable responses and ensure the complete anonymity of the participants, allowing them to feel safe and comfortable when sharing both positive and negative experiences and perspectives.

### 3.1. Category 1: Transitions in Communication and Relationships with Individuals with IDDs

*Establishing contact* was associated with uncertain perceptions and expectations among the participants about people with IDDs, and participants described fears about how they would establish contact and build professional relationships with individuals with IDDs, and what responses from these individuals would be like.

“*Before we started practice, I honestly couldn’t imagine what level of intellectual disability these individuals had. As a result, I felt more apprehensive about this practice compared to others, thinking I wouldn’t know how to approach them.*”

*Communication* was a central theme in the reflections, particularly the transition from initial uncertainty and a lack of understanding in communication to gradually recognising individual communication styles and assigning appropriate meaning to what was heard or observed.

“*At the beginning, there were a few obstacles, mainly in terms of understanding speech, but I quickly saw progress, and communication became much easier.*”

### 3.2. Category 2: Transitions in Emotions

The students described a range of *feelings*. The predominant emotions included fear, uncertainty, and feelings of inner unease. However, once they established a positive connection with people with IDDs, clinical mentors, and other healthcare professionals, adaptation occurred, bringing relief and dissipating uncomfortable feelings. The students also described pleasant emotions. Positive responses from individuals with IDDs enhanced students’ feelings of gratitude, inner satisfaction, and increased motivation for further work.

“*My fears dissipated. …each day I grew more eager to go to placement because I was able to build special relationships with many, and the users transmitted joy and a sense of belonging.*”

“*Not to mention how happy [people with IDD] are when someone mentions that we’ll take them for a walk or that it’s time for morning coffee and some sweet treats. These are the moments when one can feel joyful and proud that we chose this profession, as it’s an incredible feeling to realise you did something good for someone.*”

The reflections also focused on *empathy*. Students described a need to understand individuals with IDDs and empathise with the challenges of their everyday lives. Reactions were also described that prompted students to reflect on the concepts of quality of life and the meaning of life.

“*I didn’t feel pity for the residents [i.e., people with IDD] but rather inspiration and admiration. I realised how important the small things in life are—a greeting, a smile, a minute to listen, a short walk. These things may seem simple to us, but for the residents, they bring joy and serve as a reminder that showing compassion toward others is what truly enriches us as human beings.*”

Students found the experience of providing nursing care to be mentally taxing. They perceived it differently from previous clinical experiences, expressing a need for relief during and after the clinical placement.

“*Working with the users [i.e., people with IDD] is demanding. During the clinical placement, I missed talking with my classmates and sharing experiences, which are different from other placements.*”

### 3.3. Category 3: Transitions in Knowledge and Understanding of Care and Nursing Care

*Knowledge transitions* were related to the students’ perceptions of their lack of knowledge, skills, and competencies. With proper preparation, learning, and care experience, this uncertainty diminished, and students reflected on improved competency.

“*At first, caring for them seemed somewhat challenging because I didn’t know their [i.e., people with IDD] habits, preferences, and needs, but day by day, we got to know them better, which made caregiving easier.*”

In terms of *nursing care content*, the reflections focused on activities related to physical basic needs (such as the ability to eat, attend to personal hygiene, mobility, etc.), while descriptions of psychological and social aspects were often briefly described, centring on activities for social inclusion, companionship, and recreation. Emphasis was also placed on the time required to carry out these activities.

“*Alongside caregiving, bathing, and feeding people with IDD, we also had the opportunity to engage with them creatively, through colouring, chatting, and going for walks.*”

Students also reflected on the characteristics of the staff and environment. Staff were perceived as “*warm, understanding, friendly, wonderful, authentic, caring, patient, and accepting*”. These traits were recognised as essential for healthcare provision for individuals with IDDs. The environment, which was adapted to the users, was also identified as relevant for healthcare provision.

Students *conceptualised care and nursing care* for people with IDDs in ways that went beyond their existing understanding and experiences of healthcare. This new understanding was often described as different. This difference was also reflected in the need for tailored approaches, recognised within the concept of person-centred care.

“*The work was different from what we’re used to. The approach was more personal, and care was very individually tailored.*”

“*Care was different from what I expected because it’s necessary to encourage individuals to do as much as they can independently. These individuals are accustomed to doing what they can and want to do. If you try to do something for them, they start to resist or express discomfort or displeasure.*”

Students described how people with IDDs were provided with competent and compassionate (humanised) care. They also noted that the institution met the physical, psychosocial, and vocational potential of the individuals.

“*It seems important to me that people with IDD have a facility where they feel safe, comfortable, and that serves as both a home and a source of enjoyment. The Centre is adapted to the needs and abilities of people with IDD. It surprised and impressed me that people with IDD also have a job at the centre, which is very important because having a role in society gives everyone a sense of belonging and value.*”

## 4. Discussion

The aim of this study was to describe students’ experiences and reflections on interactions and nursing care for people with IDDs. Students’ experiences and reflections were categorised into three themes: transitions in communication and relationship with individuals with IDDs, transitions in emotions, and transitions in knowledge and understanding of care and nursing care. These categories were named to reflect “transitions”, highlighting the dynamic nature of students’ experiences. This dynamism relates to students’ responses, awareness, and active engagement in contact and nursing, as well as to the time and changes that occur around contact and the nursing care of individuals with IDDs.

In the programme for general nursing in the European Union, half of the learning hours are conducted in clinical settings across at least seven clinical fields [20,21]. Each clinical placement may thus only last a few weeks [39], requiring students to adapt quickly in terms of emotions, thoughts, behaviours, and coping with new challenges. Research indicates that initial clinical placements are often stressful for students [40,41,42] as students enter with expectations and preconceptions often misaligned with the realities of the settings and situations in which they work [43]. In the present study, it was found that students experienced uncertainty related to expectations, conceptions, contact, and nursing care for people with IDDs, initially responding with unpleasant emotions. As they developed active coping strategies to adapt to the changed conditions, these uncertainties diminished. This led to students perceiving the experience as overwhelmingly positive and essential for their future professional work and personal development. It is noteworthy that in their reflections, students expressed a deep sense of gratitude and attributed significant meaning to small events, which they felt highlighted the meaning of life. Possible reasons for this interpretation could be associated with personality traits commonly found in caregiving professions [44], as well as with students’ social awareness, particularly empathy and altruism [45] and awareness of suffering and the fragility of life [46], which was stimulated by their experience of contact and care with individuals with IDDs.

Communication was identified as a significant factor of uncertainty, with students feeling apprehensive about effectively establishing contact and communicating with people with IDDs, a finding also echoed in prior studies on nurses who do not frequently work with individuals with IDDs [4]. Students experienced a transition towards effective communication after establishing contact and nursing care for individuals with IDDs, with guidance on establishing effective communication. They became increasingly attuned to specific communication features, taking time for communication and engaging actively with people with IDDs. Such approaches to learning effective communication are one of many described in a number of studies [47,48,49].

An important finding of this study is the recognition of students’ feelings and their awareness of the need for debriefing following contact and the provision of nursing care for people with IDDs. Students wanted to reflect on their experiences with fellow students who were also on clinical placements, as well as with guidance from their teachers. Reflection and debriefing are commonly employed methods in nursing when practitioners face mentally demanding events [35,50]. A Slovenian study by Mlinar Reljić et al. [31] showed that self-reflection helps students express emotions and feelings, identify challenges, and enhance self-awareness. Some studies [30,51] suggest that reflection improves critical thinking, knowledge, and skills for future work and study. Findings from other research indicate that when students are well-prepared in advance, possess prior practical experience, are professionally socialised, and are properly mentored during clinical placements, where they also practise reflective methods, their fears, particularly anxiety and concern, are reduced, leading to faster adjustment to changes and creating appropriate emotional–social conditions for learning [42,52,53,54,55,56].

Students described discrepancies between the experiences discussed in this study and their previous clinical placements. Care and nursing were perceived holistically, focusing on the individual and integrated into the lives and routines of people with IDDs. Meanwhile, students’ descriptions of nursing interventions for individuals with IDDs concentrated predominantly on physical aspects and less on psychosocial ones. This partial contradiction between the content of nursing and a holistic understanding of care could stem from a naive, one-dimensional view of nursing content as being purely physical rather than psychosocial or spiritual. This discrepancy could also be due to the tendency of students, especially early in their education, to focus more on instrumental skills and competencies [57,58]. Furthermore, interestingly, the students did not mention the concept of interdisciplinarity in relation to holistic care. This could be explained by their focus on the physical aspects of care. Alternatively, it might indicate that they reflected solely on nursing care without considering its connection to the broader context of care for people with IDDs.

An important strength of this qualitative descriptive study was the use of written self-reflection as a method of data collection. This approach allowed us to capture participants’ personal, immediate, and unfiltered thoughts, feelings, and experiences, providing rich and authentic insights into their perceptions of caring for people with IDDs.

This is also the first qualitative study to explore nursing students’ perceptions of caring for people with IDDs in Slovenia. The findings are significant for planning clinical placement instruction, as they offer valuable insights into the challenges students face. These insights can inform the development of curricula and strategies to better support students during clinical placements.

Furthermore, this study highlights that students perceived the opportunity to engage with people with IDDs as a positive and meaningful experience. This engagement not only boosted their confidence but also helped them develop essential competencies in approaching and caring for individuals with IDD. These skills are transferable to other clinical settings, such as acute care hospitals and primary care centres.

The findings of this study are limited, particularly in terms of transferability. This study included students from a single Slovenian nursing programme, where clinical placements were of short duration and involved a variety of clinical settings caring for individuals with mild to moderate or moderate to severe IDDs, so the findings cannot be generalised as universal experiences. Due to the nature of the anonymised data collection, we were unable to attribute specific statements to individual participants and, therefore, could not further explore the potential implications of sex and age or other demographic circumstances. Another limitation is that the data analysis was based solely on students’ self-reflections, capturing manifest content only without delving into hidden, nuanced interpretations that could yield deeper levels of understanding. Furthermore, we did not perform a member check of the findings, which might have affected the credibility and transferability of the results. However, we have embraced the position that multiple realities exist and believe that our interpretation represents the most plausible understanding of the findings.

## 5. Conclusions

This study contributes to the limited collection of Slovenian research on nursing students’ self-assessment of clinical placements by providing insights and descriptions of their experiences and encounters in providing nursing care to people with IDDs. The findings indicate that students perceive contact and nursing care for individuals with IDDs as a dynamic experience, characterised by transitions in communication and relationships with individuals with IDDs, transitions in emotions, and transitions in knowledge and understanding of care and nursing care. These findings can assist in improving the planning and delivery of educational programmes at universities and in clinical settings, with the goal of preparing students effectively to provide competent, compassionate, and user-centred nursing care, as well as to foster ethical behaviour and shape positive attitudes towards individuals with IDDs.

## Figures and Tables

**Table 1:** An example of the analytical process.

Meaning Unit	Condensed Meaning Unit	Code	Subcategory	Category
Not to mention how delighted they are when someone tells them we’ll be taking them for a walk, or that it’s time for a morning coffee and sweet treats. These are the moments that make us feel both happy and proud to have chosen this profession, as there’s an incredible feeling of realising you’ve done something good for someone and made their day better.	Positive emotional responses from service users encourage a sense of inner satisfaction in students.	Inner satisfaction	Emotions	Transitions in emotions

## Data Availability

The data that support the findings of this study are available from the corresponding author upon reasonable request.
